# Primary Thyroid Lymphoma: A Rare Diagnosis Treated With Polatuzumab Vedotin

**DOI:** 10.1177/23247096241299566

**Published:** 2024-11-16

**Authors:** Rupam Sharma, Arin Orogian, Ralph Garcia-Pacheco, Tung Trang, Matthew Clarke, William Stull, Stanley Kim

**Affiliations:** 1 Department of Medicine, Kern Medical, Bakersfield, CA, USA; 2 Department of Medicine, David Geffen School of Medicine, University of California, Los Angeles, USA

**Keywords:** primary diffuse large B-cell lymphoma, chemotherapy for primary thyroid lymphoma, Pola-R-CHP

## Abstract

Primary thyroid lymphoma is one of the rare and distinct type of extra nodal lymphomas originating within the thyroid gland. It accounts for less than 5% of all thyroid malignancies and 2% of extra nodal lymphomas. It predominantly affects older adults, with a higher incidence in women. Patients typically present with a rapidly enlarging thyroid mass, accompanied by clinical symptoms of compression such as dysphagia, dyspnea, and hoarseness. Diagnosis is established through a combination of fine-needle aspiration, core needle biopsy, and advanced imaging techniques. Standard treatment involves a multimodal strategy of chemotherapy, often with the addition of radiotherapy and surgical intervention. Herein presented is the first reported case of diffuse large B-cell lymphoma treated with Pola-R-CHP.

## Introduction

Primary thyroid lymphoma (PTL) is a rare form of extranodal non-Hodgkin lymphoma that originates within the thyroid gland. Accounting for less than 5% of all thyroid malignancies and 2% to 3% of all extranodal lymphomas, PTL predominantly affects middle-aged to older women, often with a history of autoimmune thyroid disease, such as Hashimoto’s thyroiditis.^1^ The clinical presentation of PTL typically involves a rapidly enlarging neck mass, which may cause compressive symptoms such as dysphagia, dyspnea, or hoarseness.^1^

In January 2022, the POLARIX study published in the *New England Journal of Medicine* evaluated polatuzumab vedotin (pola), an anti-CD79b antibody drug conjugate (ADC) linked with monomethyl auristatin E (MMAE), in combination with rituximab, cyclophosphamide, doxorubicin, and prednisone (Pola-R-CHP), effectively replacing vincristine in the CHOP backbone. The study proved it was superior to R-CHOP among adult patients with newly diagnosed diffuse large B-cell lymphoma (DLBCL).^2,3^

To our knowledge, this is the first case reported of an 82-year-old man with diffuse large B-cell lymphoma, activated B-cell subtype (ABC, or non-GCB subtype by the Hans algorithm) of both thyroid lobes, with an international prognostic index (IPI) of 2 treated with Pola-R-CHP.

## Methods

The Institutional Review Board of Kern Medical approved this study. A retrospective review of the patient’s record was performed. A literature search was conducted on PubMed and Google Scholar using the following search terms: primary DLBCL, chemotherapy for PTL, lymphoma staging and classification, and Pola-R-CHP.

## Case Presentation

An 82-year-old man with a medical history of hypertension, diabetes mellitus II, and hyperlipidemia presented to our institution after noticing a growing mass in his left neck for 3 months. The patient stated that the neck mass had been growing slowly and asymptomatically until 3 weeks prior to his presentation. In the last 3 weeks, the patient noticed that the mass had rapidly increased in size and was associated with a pressure-like sensation aggravated by lying down on his back, and difficulty swallowing, particularly solid foods. He denied any stridor or difficulty breathing/respiratory distress. He denied any previous history of cervical irradiation or family history of thyroid cancer. Upon presentation, his vitals were blood pressure 132/82 mm Hg, heart rate 88 beats per minute, respiratory rate 20 breaths per minute, and afebrile at 36.8°C. Initial physical examination was only abnormal for a bulky neck mass from the region of the hyoid to the clavicles, which was non-tender, non-erythematous, and moderately firm to palpation. Initial laboratory workup was significant for thyroid-stimulating hormone (TSH) level of 1.456 U/mL, free T4 1.1 ng/dL, vitamin D 25 OH 34 ng/mL, serum calcium level of 12.9 mg/dL, and ionized calcium level of 1.59 mmol/L. Complete blood count was negative for leukocytosis or thrombocytopenia. Upon arrival to the emergency department, the patient underwent flexible fiberoptic laryngoscopy by otolaryngolotist which revealed a protrusion of the anterior wall of the inferior aspect of the oropharynx and marked displacement of the larynx to the left and downward. The epiglottis was seen, but the scope could not be manipulated to view the vocal cords because of the severe displacement.

**Image 1.**
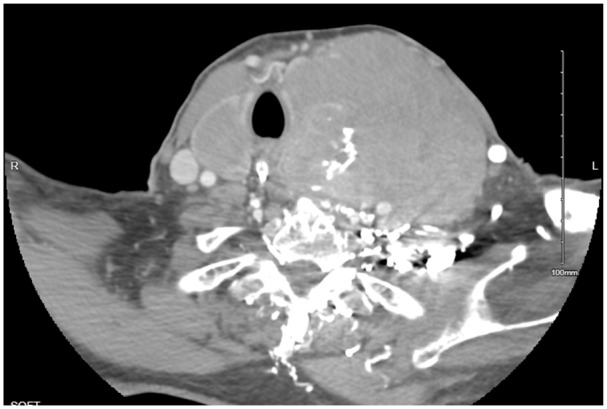
CT of the neck soft tissue with contrast: a large solid-appearing left neck mass with internal calcifications that appears similar in size up to 9.0 × 9.2 × 11 cm in size; this lesion appears to be centered within the left thyroid gland and extends into the superior mediastinum. There is a cystic nodule seen within the right thyroid lobe measuring 3.3 × 2.5 × 4.5 cm.

**Image 2.**
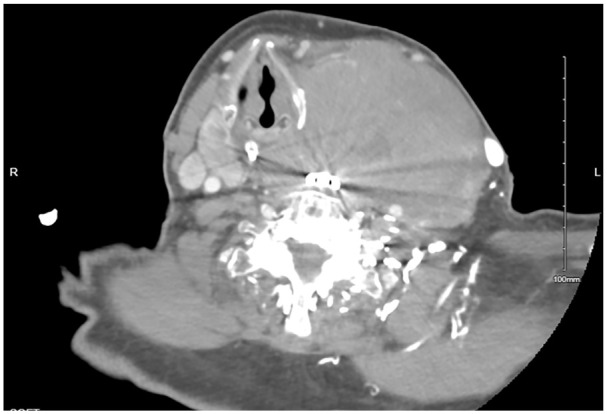
CT of the neck soft tissue with contrast: additional cystic nodule within the right thyroid lobe measuring 1.4 × 1.2 cm.

**Image 3.**
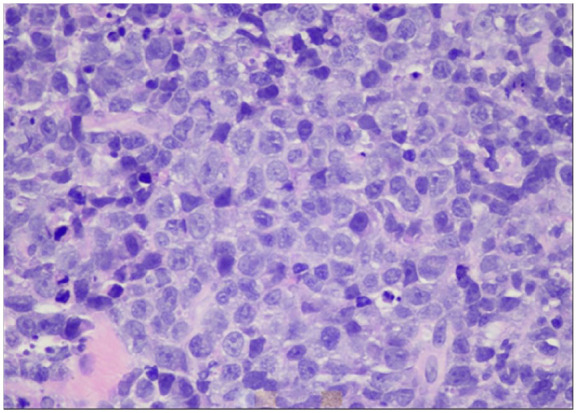
Diffuse large B-cell non-Hodgkin lymphoma, hematoxylin-eosin stain, original magnification about 400×.

**Image 4.**
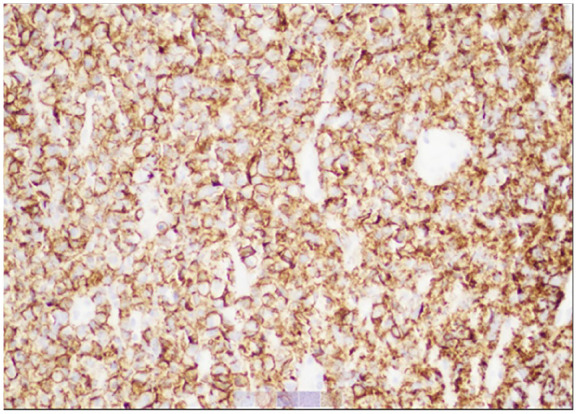
Diffuse large B-cell non-Hodgkin lymphoma and CD20-positive neoplastic cells, original magnification about 200×.

**Image 5.**
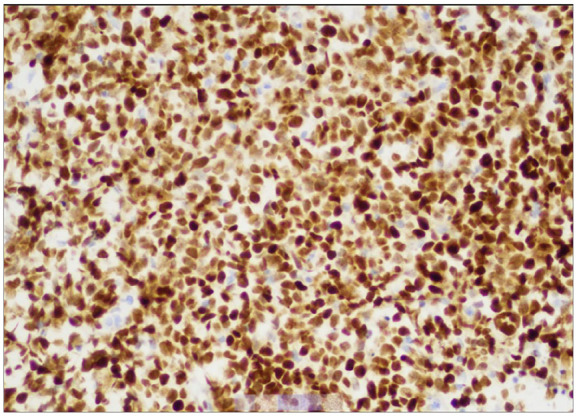
The tumor cells show strong and diffuse positive immunostaining for MUM1, original magnification about 200×.

The patient was kept nothing per oral and initiated on hydration with dextrose 5% and half normal saline at rate of 150 mL/h, calcitonin 4000 U every 12 hours, and a nasogastric tube was inserted for feeding. Computed tomography (CT) with contrast of the neck revealed a large solid-appearing left neck mass with internal calcifications measuring 9.0 × 9.2 × 11 cm; this lesion appeared to be centered within the left thyroid gland extending into the superior mediastinum. There was a cystic nodule seen within the right thyroid lobe measuring 3.3 × 2.5 × 4.5 cm, and an additional cystic nodule within the right thyroid lobe measuring 1.4 × 1.2 cm. Rightward displacement of trachea and posterior displacement of the left common carotid and internal carotid arteries related to mass effect were also noted (Images 1 and 2). Ultrasound of the head and neck was also done which revealed enlarged heterogeneous right thyroid lobe about 6.5 × 2.6 × 3.1 cm, volume of 24.7 mL; left lobe of thyroid, too enlarged to be measured; enlarged isthmus about 9.4 mm wide; heterogeneous echogenic, suspicious thyroidal masses, on right about 3.9 × 2 × 3.6 cm and 1.2 × 1.5 × 0.7 cm, on left about 10.8 × 8.9 × 7.1 cm approximately. Subsequently the patient underwent ultrasound-guided fine-needle biopsy which revealed diffuse large B-cell lymphoma, ABC subtype with an IPI of 2. Immunohistochemical analyses showed CD3 negative (small leukocytes positive), CD20 diffusely positive, CD10 negative, MUM1 positive, bcl–6 positive, cytokeratin AE1/AE3 negative, and PAX8 negative. The lack of cytokeratin and PAX8 staining excludes an anaplastic thyroid carcinoma, which was considered as part of the clinical differential. The presence of CD20 confirms a B-cell origin with lack of CD10 expression and expression of bcl-6 and MUM1 consistent with activated B-cell subtype of DLBCL (Images 3-5). Fluorescence in situ hybridization for myc, bcl2, and bcl6 rearrangements showed a bcl6 (3q27) rearrangement detected (atypical) with 1G1~2F signal pattern and 5′ partial deletion or variant rearrangement at 48% with 20% cut off.

The following day the patient was initiated on dexamethasone 20 mg IV and further workup was obtained including lactate dehydrogenase which resulted elevated to 496 U/L, intact parathyroid hormone (PTH) which resulted 7 pg/mL, and PTH-related protein which resulted elevated at 25 pg/mL. Thyroid peroxidase antibodies were negative.

The decision by the Oncology team was made to initiate patient on chemotherapy with Pola-R-CHP which consists of polatuzumab vedotin 1.8 mg/kg IV D1 (intravenous day 1), rituximab 375 mg/m^2^ IV D1, cyclophosphamide 750 mg/m^2^ IV D1, doxorubicin 50 mg/m^2^ IV D1, and dexamethasone 20 mg IV D1. The patient tolerated the chemotherapy well without any noticeable side effects or any acute reaction. Following initiation of chemotherapy, the patient’s calcium levels also normalized to 8.6 mg/dL. Over the next few days, the patient’s left neck tumor mass was found to be rapidly shrinking in size to 4 × 5 cm, fragmented, and soft, and the patient’s nasogastric tube was removed. He did not require any surgical intervention. By hospital day 9, the patient was able to tolerate oral intake without any difficulty and noticed significant improvement in his symptoms. He was discharged in stable condition with the plan for close outpatient follow-up with the heme oncologist; however, the patient was lost to follow-up.

## Discussion

Primary thyroid lymphoma is a rare and distinct type of non-Hodgkin lymphoma which originates in the thyroid gland. It accounts for less than 5% of all thyroid malignancies and approximately 2% of extra nodal lymphomas. Histologically, the most common subtype of PTL is diffuse large B-cell lymphoma (DLBCL) which accounts for greater than 50%, followed by mucosa-associated lymphoid tissue lymphomas (MALT) which accounts for 20% to 30% of cases. Further rare subtypes include follicular lymphoma with 12% of cases, Hodgkin’s disease with 7%, small lymphocytic lymphoma 4%, and Burkitt’s lymphoma of 4%.^1^ Clinically, PTL typically presents as a rapidly enlarging, painless thyroid mass which can cause compressive symptoms such as dysphagia, dyspnea, dysphonia, and hoarseness due to pressure on adjacent structures. In rare cases, approximately in 10% to 20% of cases, patients may also exhibit subjective fevers, night sweats, or weight loss.^1,4^

Chronic lymphocytic thyroiditis is a well-established factor that increases the risk of developing PTL by 40% to 80% when compared with the general population.^5,6^ According to some publications, over 90% of PTL cases are associated with Hashimoto’s disease, which was not the case or the patient had no prior history of any thyroid disease.^7^ Primary thyroid lymphoma is more commonly seen in female patients with female-to-male ratio of 3-4:1 and usually presents in the seventh decade of life, with mean being 65 years of age.^1^

The Ann Arbor staging criteria, which was originally developed for Hodgkin lymphoma, can also be applied to PTL to assess the extent and spread of the disease. This staging system classifies lymphoma into 4 stages: Stage IE is defined as lymphoma limited to the confines of the thyroid gland, stage IIE represents spread beyond the thyroid to regional lymph nodes, stage IIIE represents involvement of lymph nodes on both sides of the diaphragm, and stage IVE indicates systemic dissemination.^1,8^ Although PTL is extremely rare, it should always be included in the differential diagnosis of a rapidly growing goiter and/or thyroid nodules.^8^ As of this date, no definitive treatment has been suggested for successfully treating this disease; however, several studies have suggested the use of chemotherapy, commonly CHOP (cyclophosphamide, doxorubicin, vincristine, prednisone) in addition to rituximab which was approved in 2006 for patients with extra nodal lymphomas.^5,8^

Definitive diagnosis requires fine-needle aspiration cytology (FNAC) and biopsy as clinical history; physical examination, thyroid function tests, or imaging alone are not enough to diagnose PTL.^1,4^ When FNAC is not diagnostic and there is a high degree of suspicion, a core biopsy or any incisional biopsy or even a thyroidectomy may be required; however, core biopsies have been demonstrated to diagnose and classify 95% of lymphomas.^1,9^ Once the diagnosis has been established, further studies are warranted for further staging of the patient including CT of the head, neck, chest, abdomen, and pelvis. The therapeutic approach to PTL is controversial due to its rarity, currently most authors advocate for a multidisciplinary approach.

Our patient showed the common clinical presentation with recent sudden onset of a painless, large neck mass causing compressive symptoms of dysphagia, dyspnea, dysphonia, and hoarseness. Histology and immunochemistry revealed DLBCL with positivity of CD20 and a high proliferation index. For nearly 2 decades, attempts to change to frontline treatment for DLBCL have remained unsuccessful, despite efforts to extend the duration or intensity of treatment and adding targeted agents to the current R-CHOP (rituximab, cyclophosphamide, doxorubicin, vincristine, prednisone/prednisolone) regimen.^2^ The POLARIX study, published in the *New England Journal of Medicine* in January 2022, evaluated the combination of an anti-CD79b ADC, polatuzumab vedotin (pola) linked with MMAE, in combination with rituximab, cyclophosphamide, doxorubicin, and prednisone (Pola-R-CHP) effectively replacing vincristine in the CHOP regimen. Pola-R-CHP proved superior to R-CHOP in adult patients with newly diagnosed DLBCL with an IPI of 2 or higher.^2,3^ In our case, the patient was initiated on chemotherapy with Pola-R-CHP which the patient tolerated very well without any side effects.
